# TUHAD: Taekwondo Unit Technique Human Action Dataset with Key Frame-Based CNN Action Recognition

**DOI:** 10.3390/s20174871

**Published:** 2020-08-28

**Authors:** Jinkue Lee, Hoeryong Jung

**Affiliations:** Department of Mechanical Engineering, Konkuk University, 120 Neungdong-ro, Jayang-dong, Gwangjin-gu, Seoul 05029, Korea; ejk0502@naver.com

**Keywords:** gesture recognition, action recognition, convolutional neural network, taekwondo, poomsae, human action dataset

## Abstract

In taekwondo, poomsae (i.e., form) competitions have no quantitative scoring standards, unlike gyeorugi (i.e., full-contact sparring) in the Olympics. Consequently, there are diverse fairness issues regarding poomsae evaluation, and the demand for quantitative evaluation tools is increasing. Action recognition is a promising approach, but the extreme and rapid actions of taekwondo complicate its application. This study established the Taekwondo Unit technique Human Action Dataset (TUHAD), which consists of multimodal image sequences of poomsae actions. TUHAD contains 1936 action samples of eight unit techniques performed by 10 experts and captured by two camera views. A key frame-based convolutional neural network architecture was developed for taekwondo action recognition, and its accuracy was validated for various input configurations. A correlation analysis of the input configuration and accuracy demonstrated that the proposed model achieved a recognition accuracy of up to 95.833% (lowest accuracy of 74.49%). This study contributes to the research and development of taekwondo action recognition.

## 1. Introduction

Vision-based action recognition is an important research topic in the domain of human action recognition. Many action recognition studies have adopted vision sensors because of the rich information they provide, despite their limited field of view and sensitivity to lighting changes [1]. Recent advances in artificial intelligence technology and vision sensors have promoted vision-based action recognition for various applications, such as education [2], entertainment [3,4], and sports [5,6,7,8,9,10,11,12]. Various studies have proposed novel algorithms [13,14,15,16,17,18,19,20,21,22,23] or established datasets [1,24,25,26,27] for vision-based action recognition.

Sports is one sector with significant adoption of vision-based action recognition. Several action recognition algorithms have been proposed for sports, including soccer [5], golf [6,7], tennis [8,9,10], table tennis [11], and baseball [12]. Vision-based action recognition systems have been applied to provide quantitative scoring standards or systems to assist referees. However, relatively few studies have investigated the application of vision-based action recognition to martial arts. The actions of martial arts are significantly more rapid than normal movements, which complicates the acquisition of continuous actions with sufficient clarity and without the loss of inter-frames. In addition, martial arts poses require rapid posture changes and an extreme range of action, which complicates the application of existing human action databases and software libraries developed for normal actions [28]. Many studies have used skeleton data obtained with RGB-D sensors for action recognition [16,19,20,21,22,23,29,30]. Such skeleton data provide information on the locations of joints over time, which can be utilized for action recognition [29]. In the literature, skeleton data have been combined with machine learning algorithms such as the convolutional neural network (CNN) [16,19,21,22,23] or recurrent neural network [29,30] for human action recognition. However, using such data for martial arts with a wide range of movements, and extreme and rapid actions is difficult because the RGB-D sensors have low skeleton extraction accuracy for those actions [31,32]. Several studies on action recognition for martial arts have sought to address these challenges. Zang et al. [32] and Soomro et al. [33,34] proposed action recognition methods that can be applied to martial arts as well as general sports and routine movements, and Heinz et al. [35] attached sensors to users for kung fu action recognition. Salazar et al. [36] proposed using Kinect to automate the evaluation process of martial arts forms, and Stasinopoulos and Maragos [37] proposed an algorithm based on the historiographic method and hidden Markov model for martial arts action recognition from videos. However, while studies focused on typical human action recognition could leverage various action datasets and recognition algorithms, previous studies on martial arts action recognition had no public datasets and recognition algorithms specific to martial arts that were available to them.

Taekwondo is an official Olympic sport consisting of two major categories: gyeorugi and poomsae. Gyeorugi is a type of full-contact sparring between opponents that uses a quantitative and accurate electronic scoring system to facilitate objective judgment. In contrast, poomsae is a form competition where a single competitor arranges basic attack and defense techniques in a certain order. Unlike *gyeorugi*, which has quantitative and accurate scoring, poomsae is scored by judges. Except for certain penalties such as timeouts and borderline violations, the judging is subjective and qualitative. In addition, situational constraints mean that judges must often evaluate multiple competitors simultaneously. Therefore, issues have arisen over scoring fairness and consistency for poomsae, not only in competitive events but also in promotion examinations.

Several studies have focused on vision-based action recognition for taekwondo. De Goma et al. [28] proposed a taekwondo kick detection algorithm with skeleton data preprocessing. Choi et al. [38] proposed a system for remotely scoring poomsae by comparing actions acquired from multiple vision sensors with corresponding reference actions. Seo et al. [39] suggested a Poisson distribution-based recognition algorithm that uses one-dimensional spatial information extracted from image sequences as its input. Kong et al. [40] extracted taekwondo actions from a broadcast competition video and classified them with high accuracy by applying a support vector machine and defining a taekwondo action as a set of poses. However, these studies had limitations such as low accuracy despite a complex recognition system [38], restricted applicability arising from limited movements [28], and vulnerability to the movement of subjects because of the exclusive use of histogram images [39]. Despite a high recognition accuracy rate, Kong et al.’s method [40] requires two training and recognition models each for a single frame and action classification process, and the pose order needs to be defined for all action classes. This results in unique actions that greatly influence the behavior definition. Thus, if an action is performed in a different pose order (e.g., a transitional action), the recognition accuracy can decrease [40]. Furthermore, the task of labeling 16 poses requires a significant amount of time and resources.

To address these problems, a simpler system, dataset optimized for taekwondo, and method to minimize manual intervention in the action recognition process are required. In this study, the Taekwondo Unit technique Human Action Dataset (TUHAD see Supplementary Materials) was compiled for taekwondo action recognition, and a key frame-based action recognition algorithm was proposed. The proposed algorithm was validated through an accuracy analysis for various input configurations. The main contributions of this study are as follows:

1. TUHAD was constructed for application to taekwondo action recognition, and it includes the representative unit techniques of taekwondo. All actions were performed by taekwondo experts and were collected in a controlled environment for high reliability.
2. A key frame-based CNN architecture was developed for taekwondo action recognition. The accuracy of the proposed model was analyzed according to various input configurations regarding the image modality, key frame position, camera view, and target action to determine the optimal settings for data gathering and action recognition based on taekwondo-specific characteristics.

## 2. Materials and Methods

**Figure 1:**
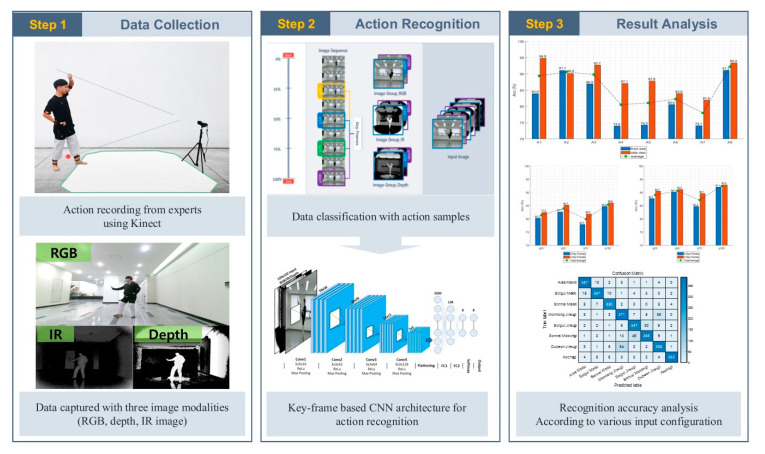
Overall process of the proposed action recognition method.

Figure 1 shows the proposed method for taekwondo action recognition, which includes data collection, action recognition, and accuracy analysis. For data collection, image sequences of the taekwondo unit techniques were acquired to establish TUHAD. All unit techniques in TUHAD are from the first three chapters of Poomsae Taegeuk (i.e., set of forms), and they were performed by professional taekwondo experts. A mono RGB-D camera was used to acquire the multimodal image sequences. The key frame-based CNN action recognition architecture was trained with TUHAD. The accuracy of the recognition model was then tested with various input configurations to determine the optimal configuration for taekwondo action recognition.

### 2.1. TUHAD: Taekwondo Unit Technique Human Action Dataset

TUHAD consists of approximately 100,000 image sequences of eight basic unit techniques of taekwondo performed by 10 experts. All actions were recorded from the front and side views. The experimental protocol was approved by the Konkuk University Institutional Review Board (7001355-202004-HR-375).

#### 2.1.1. Subjects

Ten professional taekwondo poomsae demonstrators (or equivalent) of various heights and body types participated in the experiment to collect action data for TUHAD. No clothing restrictions were imposed on the subjects. The subjects were designated as T1–T10.

#### 2.1.2. Data Modalities

Microsoft Kinect v2 was used to collect image sequences and capture RGB, depth, and infrared images concurrently. The depth and infrared images that were input to the dataset had a raw resolution of 512 × 424 pixels. The RGB images were down-sampled from 1980 × 1080 pixels to 786 × 424 pixels to reduce memory requirements and match the horizontal resolution of the depth and infrared images. All images were captured from two different camera views: front and side. The front-view images were filmed so that the subject approached the camera from the starting point, and the side-view images captured the movements of the subject from the starting point to the right of the camera. In total, 99,982 image frames were captured for each image modality and input to TUHAD.

#### 2.1.3. Capture Setup

**Figure 2:**
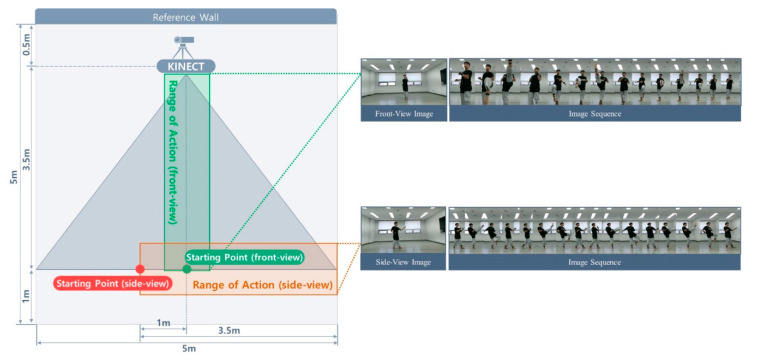
Capture space and setup for front and side view images.

**Figure 3:**
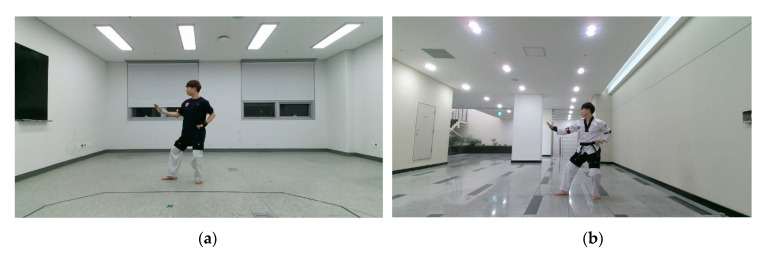
Two image capture environments: (**a**) 70% of the dataset images recorded here; (**b**) the remaining 30% recorded here.

Figure 2 shows the setup for capturing actions from the front and side views with a single RGB-D sensor. During data collection, each subject performed eight unit techniques in a predefined order while the sensor recorded the action from a fixed location. The capture space was a 5 × 5 m square set up so that the subjects would remain within sensing range considering the range of movement of the unit techniques. The camera was located 0.5 m from the center of the reference wall. To capture the front- and side-view images, the subjects performed the same actions twice in different directions. For the front-view image capture, a subject performed an action while moving towards the sensor from the starting point located 4 m away from the center of the reference wall. For the side-view image capture, the subject moved to the other starting point, which was 1 m to the left of the front-view starting point, and repeated the same action while moving parallel to the reference wall. All movements were recorded indoors and under artificial lighting. To reduce the influence of background elements on the training process, 30% of the subjects were recorded in another environment under different background and lighting conditions, as shown in Figure 3.

#### 2.1.4. Action Classes and Action Samples

**Figure 4:**
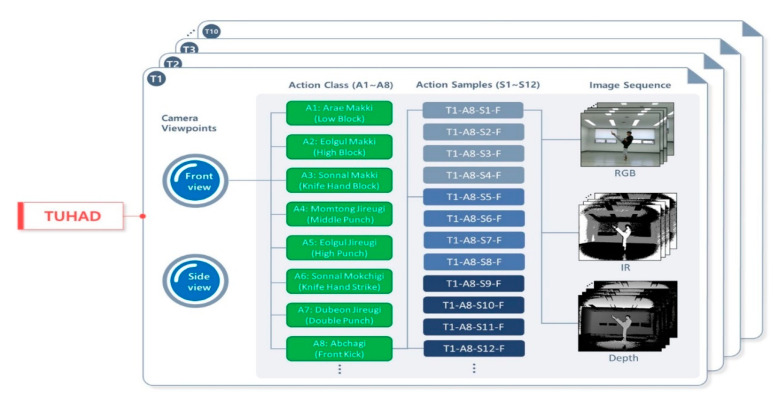
Hierarchy of action classes and action samples in Taekwondo Unit technique Human Action Dataset (TUHAD). Each hierarchy level is repeated at a higher layer.

**Figure 5:**
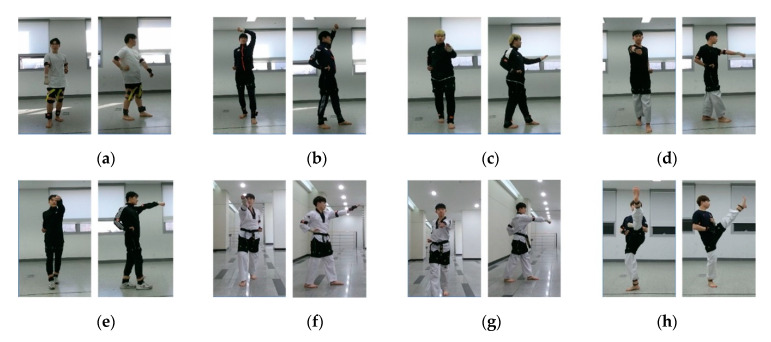
Eight taekwondo actions in TUHAD: (**a**) arae makki (low block, A1), (**b**) eolgul makki (high block, A2), (**c**) sonnal makki (knife hand block, A3), (**d**) momtong jireugi (middle punch, A4), (**e**) eolgul jireugi (high punch, A5), (**f**) sonnal mokchigi (knife hand strike, A6), (**g**) dubeon jireugi (double punch, A7), and (**h**) abchagi (front kick, A8). All actions were recorded with two camera views.

**Figure 6:**
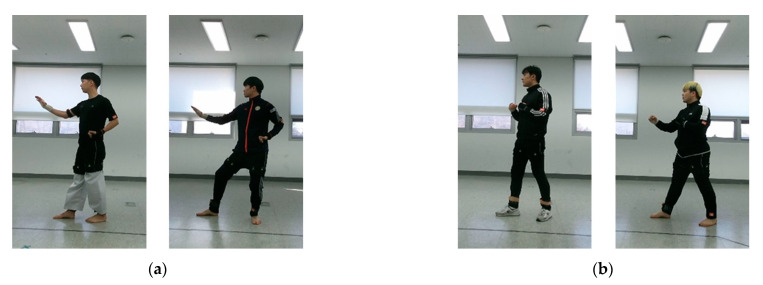
Differences between the subjects when performing unit techniques: (**a**) standing postures for apseogi (left, walking stance) and dwisgubi (right, back stance); (**b**) transitional actions of abchagi (front kick, A8).

Figure 4 shows the hierarchical structure of TUHAD. The dataset is classified first by subject (T1–T10) and then by camera view (F, S) in the subsequent child node. Each view subset comprised eight action classes (A1–A8) corresponding to the eight unit techniques of the first three chapters of Poomsae Taegeuk. Figure 5 shows front and side views of a representative pose for each action. These unit techniques are the basics of taekwondo and comprise three makki (block), three jireugi (punch), one chigi (strike), and one chagi (kick). For each subject, one action class set comprised 12 action samples (S1–S12), each representing a set of consecutive images for one-unit technique. Each action sample consisted of three different image modalities: RGB, depth, and infrared. For data collection, four action samples were captured in a single take as the subject performed the same action continuously four times while moving forward from the starting point. This procedure was repeated three times, with the subject returning to the starting point each time, to acquire 12 action samples for one action class. There were no restrictions on the stride or standing posture to allow for variability within the actions. Therefore, different subjects executed the same unit technique with variations in the standing posture, position on the screen, and transitional actions. Figure 6a shows the differences between the standing postures of subjects T2 and T4 performing action class A3, and Figure 6b shows the difference between subjects T5 and T3 performing A8. Some of the unit techniques were mistakenly performed four times, and the additional samples were added to the dataset in the same manner as the other data samples without any modification. However, action samples (e.g., A3 by subject T5) that were performed incorrectly were excluded from the dataset. There should normally be 96 samples for each camera view (eight action classes × 12 action samples), but these mistakes are the reason for the irregularities in the number of samples among subjects as given in Table 1.

#### 2.1.5. Data Organization

**Figure 7:**
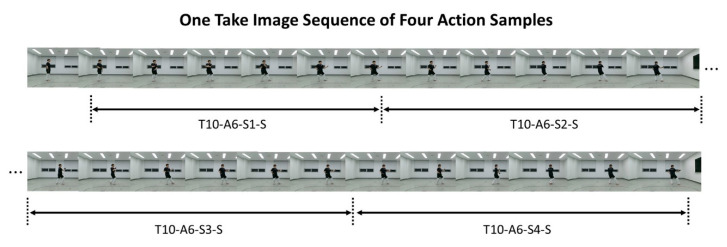
Data organization process: take image sequence including four action samples. The start and end frames of each action sample are marked to distinguish individual action samples.

An action class captured in a single take was manually divided into four action samples, where the beginning and ending frames were marked for each unit technique as shown in Figure 7. Three annotators indexed and crosschecked the data several times to ensure data reliability. First, each annotator individually searched the frames corresponding to the beginning and end of an action while watching the image sequence frame by frame and recorded the frame number in a separate document. Second, the initial indexing results produced by each annotator were compared, and the correct indexing result was selected by majority. If there was no majority indexing result, then all annotators searched the beginning and end frames of an action together again, and a final agreement was derived through discussion. Third, the final indexing results were carefully verified one more time by the annotators to ensure the integrity of the database. Finally, the action samples separated from the long take were placed in hierarchical data folders, as shown in Figure 4.

### 2.2. Key Frame-Based Action Recognition with CNN

In studies on vision-based action recognition, image frames extracted from the action sequence (i.e., static pose images) have been used to recognize complex human actions [17,40,41]. Kong et al. [40] defined 16 preset poses and assumed that all taekwondo actions could be represented as a combination of these poses. Although their method achieved a mean classification accuracy of up to 91.3%, the recognition rate was relatively low for transitional actions (i.e., movements connecting different actions or poses). This was because their method strongly depended on manual adjustments to set each pose combination. In this study, a key frame-based action recognition algorithm is proposed that is based on a CNN architecture, which is known to be effective for image pattern recognition. The key frames are the set of images representing the distinct features of a target action and are used as the input for the CNN classifier. Regardless of differences in the action speed and technique of subjects, poses corresponding to the moment as a proportion of the entire action sequence can be assumed similar because unit techniques need to be performed in certain ways in taekwondo. Under this assumption, key frames can be selected automatically according to a predefined proportional position with the entire action sequence. The CNN classifier was customized for key frame-based taekwondo action recognition, and the optimal key frame positions were determined through recognition accuracy analysis.

#### 2.2.1. CNN Input Data

**Figure 8:**
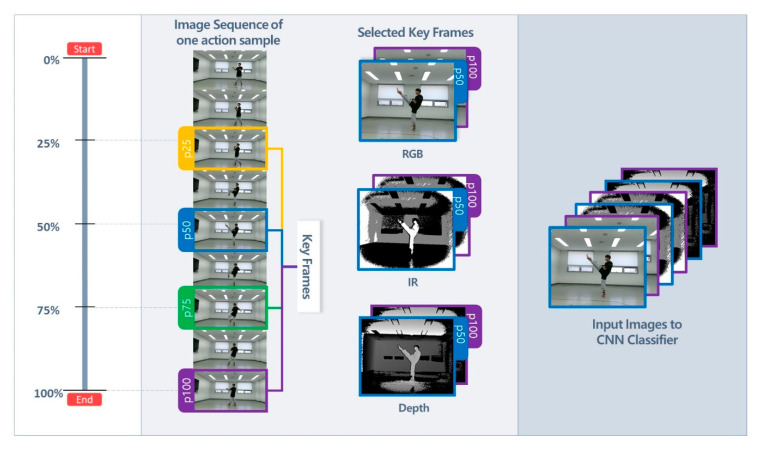
Process of constructing the key frame-based multimodal input images for the convolutional neural network (CNN) classifier.

One set of multichannel images was used as the input for the key frame-based CNN classifier. The size of each input image was set to 120 × 120 pixels, and the images used for training and testing were resized to match. All RGB images needed their edges cropped before resolution adjustment because they had different resolution ratios compared to the depth and IR images. As shown in Figure 8, the input images comprised a stack of image groups of a specific data modality, and each image group comprised two to four key frames extracted from the action samples of the unit techniques. Various input data configurations were obtained for the three independent variables: image modality, key frame position, and camera view. The dependence of accuracy on the input data configuration was analyzed as follows.

*Image modalities*: Action samples of the unit techniques were acquired with three different image modalities: RGB, depth, and infrared. Previous studies have employed methods that superimpose different image modalities into one multichannel image to improve the classification and action recognition performances [41,42]. In this study, various modalities of key frame images were simply stacked into one multichannel image, which was used as the input for the CNN classifier. Seven input image configurations were created from different combinations of the three modalities. The seven input configurations were used to analyze their correlation with the recognition accuracy, and the optimal configuration was selected for subsequent analyses.

*Key frames*: A minimum of two images was required for accurate recognition because all unit techniques consist of changes in poses over time. Several key frames were defined and used to extract multiple images from one action sequence. The images located at a constant ratio were extracted based on the start and end of an image sequence. The first image of each action image sequence was designated as the 0% position, and the last image was taken as the 100% position. The frame images located at 25%, 50%, 75%, and 100% of the image sequence were then defined as key frames and were labeled as p25, p50, p75, and p100, respectively. There was no designation of p0 because the first image of the action sequence was intentionally excluded in consideration of the characteristics of taekwondo: even for the same technique, the initial pose and movement depend on the previous technique. For example, arae makki (low block, A1), which begins in the preparation stance and is performed following other techniques, does not always have the same initial stance. Therefore, the initial image frame of actions did not have generality, and only the other key frame positions were used. Two to four key frames were used as the input data for the different training models. This yielded six input configurations with two key frames, four input configurations with three key frames, and one input configuration with four key frames. The correlation between the number of key frames and recognition accuracy was analyzed, and the optimal key frame containing the most important features of an action was identified.

*Camera views and action classes*: The actions of unit techniques were captured in two different viewpoints: front and side viewpoints. All unit techniques were sampled independently in each view. These separate action samples were used to analyze the variation in the recognition accuracy as a function of the camera view. In addition, the accuracy for each action class was analyzed to detect taekwondo characteristics that could be utilized in action recognition.

#### 2.2.2. CNN Architecture

**Figure 9:**
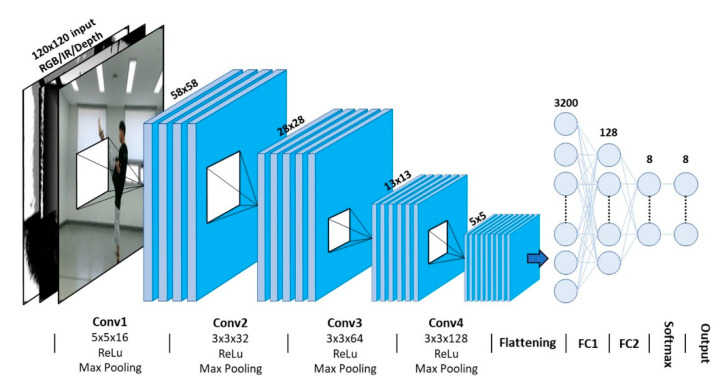
Proposed architecture of the key frame-based CNN.

The CNN architecture for action classification was constructed by considering existing CNN-based action recognition architectures [11,16,41,43]. In total, four convolutional layers were used with 16 5 × 5 filters, 32 3 × 3 filters, 64 3 × 3 filters, and 128 3 × 3 filters. All hidden layers used the rectified linear unit (ReLU) as the activation function. Max pooling was performed after each convolutional layer over 2 × 2 spatial windows with a stride of 2. After the data were passed through two fully connected layers, a softmax layer was applied to obtain the classification score. Figure 9 shows the proposed CNN architecture for taekwondo action recognition, and Table 2 presents the number of training parameters and the output shapes of each layer.

#### 2.2.3. Training Steps

Parametric training was conducted with 90% of the samples, and the remaining 10% were used to test the trained model. The training and test samples were selected randomly from the entire dataset that contains action samples of all subjects. During the training process, fivefold cross-validation was used to obtain the generalization accuracy of the predictive models. All data were flipped left and right during the training process because taekwondo is divided into right-handed and left-handed actions, even for the same technique. However, these actions have perfect bilateral symmetry, and there are no other differences. Therefore, the images were flipped to double the number of training samples. The ADAM optimizer was applied to train the parameters [44]. The maximum number of epochs was set to 200. The cross-entropy loss was used as the objective function. Table 3 summarizes the characteristics of the training and test data.

For a multichannel input image set of *n* samples:

- Flipped samples: 2*n*;
- Number of test samples: 2*n* × 0.1;
- Number of validation samples: 2*n* × 0.9 × 0.2;
- Number of training samples: 2*n* × 0.9 × 0.8.

## 3. Results

The experimental results and recognition accuracy of the proposed method for the various input configurations are presented here. The recognition accuracy was analyzed with regard to the image modality, key frames, camera view, and action class.

### 3.1. Image Modalities

**Figure 10:**
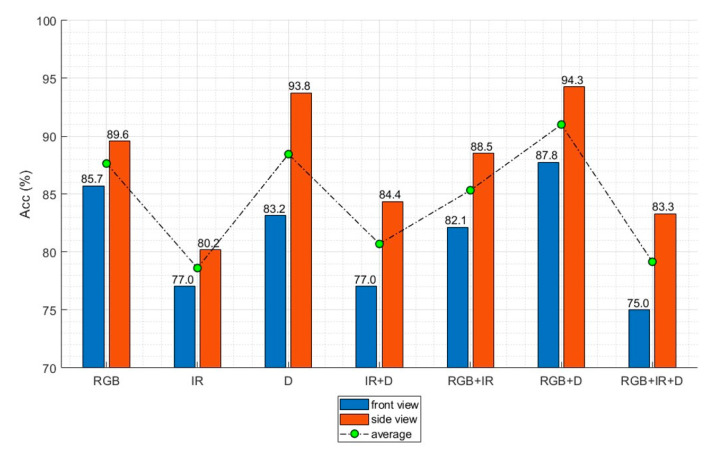
Recognition accuracy according to the image modality.

The recognition accuracy was evaluated for seven input configurations comprising various combinations of the three image modalities, as given in Table 4. All four key frames (i.e., p25, p50, p75, and p100) were used for the multimodal input data to prevent them from influencing the accuracy. Table 4 and Figure 10 present the accuracy of the proposed method according to the input image modalities. The accuracy was measured by using test samples that were not used during the training process. To evaluate the recognition accuracy, 196 and 192 test samples were used for the front and side views, respectively. Combining the RGB and depth image modalities provided the highest accuracy for both the front and side views at 87.7% and 94.2%, respectively. The lowest accuracy was obtained by using only the infrared image modality for both the front and side views at 77.0% and 80.2%, respectively.

### 3.2. Key Frames

**Figure 11:**
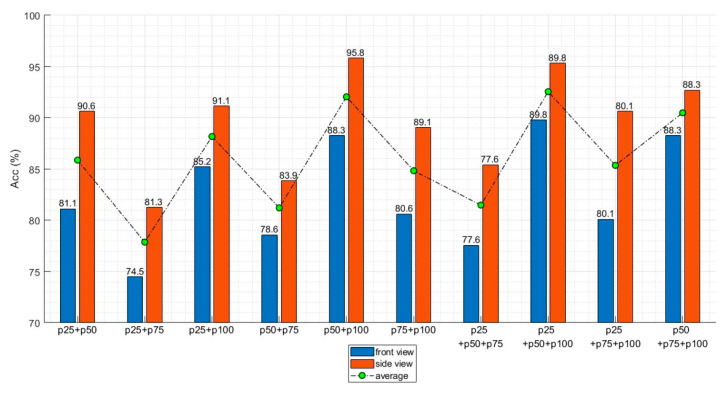
Recognition accuracy according to the selected key frames.

The effect of each key frame on the recognition accuracy was analyzed. In total, 11 input configurations were created from various combinations of the four key frames (i.e., p25, p50, p75, and p100). Table 5 and Figure 11 present the key frame combinations and obtained recognition accuracy for the front and side views. The recognition accuracy was evaluated with 196 and 192 test samples for the front and side views, respectively. The highest recognition accuracy with two key frames was obtained by combining p50 and p100 at 88.2% and 95.8% for the front and side views, respectively. With three key frames, the highest accuracy was obtained for the combination of p25, p50, and p100 at 89.7% and 95.3% for the front and side views, respectively. The combinations with the highest accuracy were p25, p50, and p100 for the front view and p50 and p100 for the side view.

**Figure 12:**
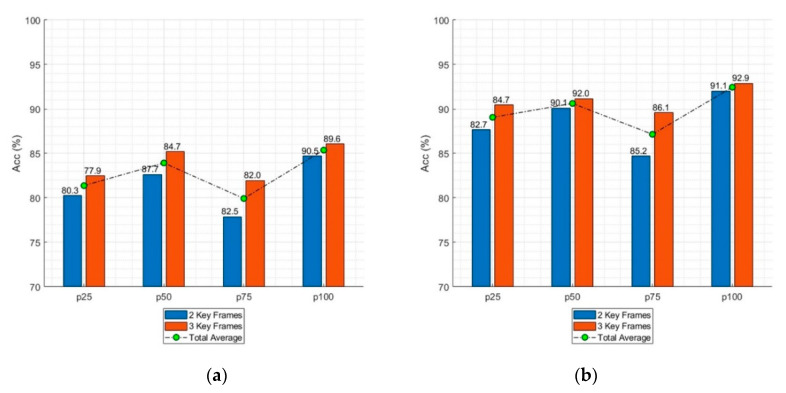
Average accuracy by key frames for each camera view: (**a**) front and (**b**) side.

Table 6 and Figure 12 present the average recognition accuracy for a specific key frame. In Table 6, the accuracy of each key frame indicates the average accuracy of all combinations that includes this key frame. If p25 is taken as an example, the accuracy for two key frames was calculated by averaging the accuracies of (p25 + p50), (p25 + p75), and (p25 + p100). The accuracy for three key frames was calculated by averaging the accuracies of (p25 + p50 + p75), (p25 + p50 + p100), and (p25 + p75 + p100). The accuracies of the other key frames were calculated in the same manner. The highest average accuracy was obtained with p100, and the lowest average accuracy was obtained with p75.

### 3.3. Camera Views and Action Classes

**Figure 13:**
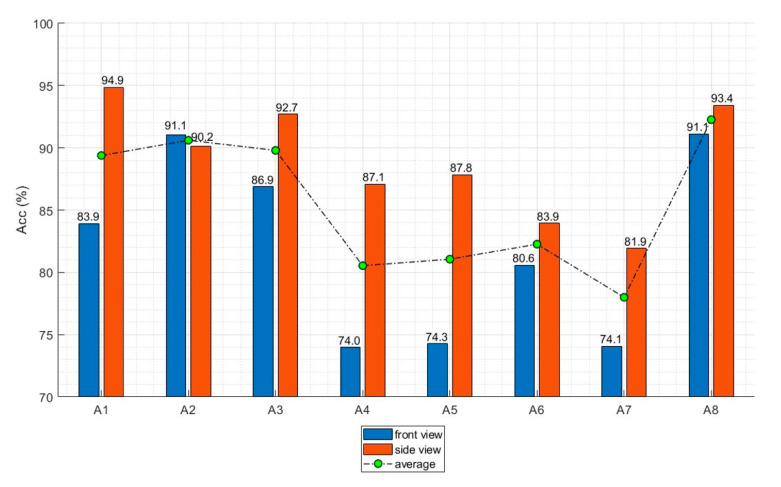
Recognition accuracy according to the action class: front view (blue) and side view (red).

**Figure 14:**
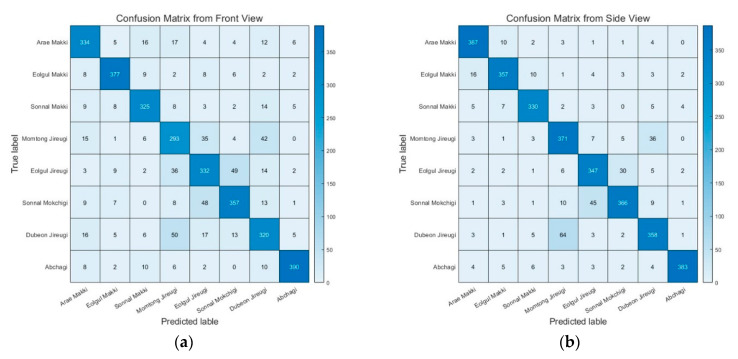
Confusion matrices of the cumulative test results: (**a**) front view and (**b**) side view.

Table 7 presents the recognition accuracy for each action class and camera view. As indicated in Table 4 and Table 5, the accuracy was about 2–10% higher with the side view than the front view for almost all analyses. The same trend can be observed in Table 7. The average accuracy was 88.8% with the side view and 81.8% with the front view. This tendency was confirmed for each unit technique except for A2. With regard to action classes, the three jireugi actions (A4, A5, and A7) and one chigi action (A6) had lower accuracies than the others, as shown in Figure 13. Figure 14 shows the confusion matrices for the action classification. The most frequently confused actions were momtong jireugi (middle punch, A4) versus dubeon jireugi (double punch, A7) and sonnal mokchigi (neck-high knife hand strike, A6) versus eolgul jireugi (high punch, A5).

### 3.4. Backgrounds 

Table 8 shows the effect of the background to the recognition accuracy. TUHAD was captured in two different backgrounds as shown in Figure 3. In total, 70% of images were captured in a background shown in Figure 3a and remaining 30% of images were captured in the other background shown in Figure 3b. To compare the recognition accuracy regarding the backgrounds, the accuracies were evaluated for five sets of training and test samples having different background configurations as shown in Table 8. The recognition accuracy of the model trained with the samples captured in the background A and tested with the images captured in the background B was 44.2% and 47.4% for the front and the side viewpoint, respectively. On the contrary, when training was conducted with images captured in the background B and tested with the images captured in the background A, the accuracy was 30.0% and 30.7% for front and side viewpoints, respectively. These cases, in which training and testing were conducted with images of different backgrounds, showed significantly lower accuracy than the model trained with the images of both backgrounds. However, there were no significant differences in recognition accuracy for the cases in which the training was conducted with the images of both backgrounds. These results imply that the problem of such overfitting may arise if the training data is captured in a specific environment.

### 3.5. Comparison with Previous Datasets and Recognition Algorithms 

In this paper, we proposed the dataset and the recognition algorithm specialized for taekwondo action recognition. This section compares the proposed dataset (TUHAD) and the recognition algorithm with the existing datasets and recognition algorithms, respectively. Table 9 shows the comparison of the proposed dataset with the previous one containing sports and martial arts actions. The datasets were compared regarding the field, recording environment, modality, number of action classes, samples, and subjects. The comparison with previous datasets demonstrates that the proposed dataset contains sufficient action samples, subjects, and classes with various image modalities for a relatively narrow field of taekwondo compared to the previous datasets.

The performance of the proposed key frame-based taekwondo action recognition algorithm was quantitatively compared with the previous algorithms [39,40] applied for taekwondo action recognition regarding the recognition accuracy and computation time required for the action recognition. Since the previous algorithms were specialized for their own datasets, it was difficult to apply the algorithms to TUHAD. Thus, the previous algorithms have been slightly modified to fit into our dataset while maintaining their methodology. RGB image of key-frame p100, which shows highest recognition accuracy among the key frames, was given as the input to the previous algorithms receiving single input image. The recognition accuracy of the proposed method was evaluated as the average recognition accuracy of the key frame p100 shown in Table 6 for fair comparison. The computation time was measured in the machine equipped with NVDIA GeForce RTX 2060 GPU, Intel i7-9700 CPU, and 64 GB Ram. Table 10 shows the results of performance comparison. For the recognition accuracy, the proposed method was 24.7% and 8.5% higher than that of the previous method [40]. For the computation time, the proposed method also showed the superior result which was two times faster than the previous method [40].

## 4. Discussion

Implications and insights obtained from the analysis of how the various input configurations affected the recognition accuracy are presented here. They are pertinent to understanding the important features of taekwondo action recognition and can be utilized for improved accuracy and reliability in further research.

### 4.1. Image Modalities

The analysis of the input image modalities indicated that combining RGB and depth images provided the highest recognition accuracy. In contrast, image modality combinations that included infrared images achieved relatively low accuracy compared to the other combinations. For example, adding infrared images to the RGB and depth images lowered the recognition accuracies by 14.5% and 11.6% for the front and side views, respectively. This result is interesting because the accuracy declined even though more information was given to the action classifier. The results confirmed that simply stacking image modalities is a feasible approach to improving the recognition accuracy, rather than using various image preprocessing techniques or creating new types of images [11,40,41].

### 4.2. Key Frames

The proposed action recognition algorithm uses 2–4 key frames extracted from a sequence of images of the target action. Using only the key frames rather than the entire image sequence allows the proposed algorithm to achieve acceptable recognition accuracy while maintaining computational efficiency. The correlation between the recognition accuracy and different combinations of key frames (p25, p50, p75, and p100) were analyzed. As shown in Figure 11, the highest accuracy for the side view was achieved by combining p50 and p100, followed by combining p25, p50, and p100. For the front view, the highest accuracy was achieved by combining p25, p50, and p100, followed by combining p50 and p100. This implies no relationship between the number of key frames and recognition accuracy. If a critical key frame presents a distinct feature of an action, sufficient recognition accuracy can be achieved even without using the information from all frames. This conclusion is further supported by the fact that the accuracy was higher with p50 and p100 than with all of the key frames for both the front and side views. However, the position of the key frame greatly affected the recognition accuracy, as demonstrated in Table 6 and Figure 13. Regardless of the camera view, p100 provided the highest recognition accuracy followed by p50, p25, and p75. This implies that the last pose of a taekwondo action contains its most distinct features.

### 4.3. Camera Views and Action Classes

The recognition accuracy was 2–10% higher with the side view than with the front view for all action classes except for A2, as shown in Figure 13. This was expected because of the nature of taekwondo actions, in which many movements are made while looking forward. When a limb is extended toward the front (e.g., jireugi (punch)), extracting shape information for the arms and legs from the front view is difficult. Therefore, action features can be observed more easily from the side than from the front. Four action classes (three jireugi (middle punch, A4; high punch, A5; double punch, A7) and one chigi (neck-high knife hand strike, A6)) had lower recognition accuracies than the other classes for both the front and side views. For the four action classes, a common feature was the arms were swung while straight. In particular, the recognition accuracy was below 80% for the three jireugi actions from the front view, which can be attributed to their high visual similarity. As discussed previously, determining the angles of the arms from the front view was difficult. In addition, the fist may overlap the torso, which makes it difficult to recognize. In contrast, these techniques can be distinguished more clearly from the side view because the front of the arm does not overlap with the torso. This conclusion is supported by the fact that the recognition accuracy for the jireugi actions was relatively high from the side view.

The low recognition accuracy for the front view due to visual similarity is also shown in Figure 14. The most frequently confused movements were momtong jireugi (middle punch, A4) versus dubeon jireugi (double punch, A7) and sonnal mokchigi (neck-high knife hand strike, A6) versus eolgul jireugi (high punch, A5). A4 and A7 only differ in the number of punches performed, and the punching action and target point are the same; thus, these two actions result in the same pose at p100. A5 and A6 are aimed at the face and neck, respectively, so they have similar heights for the hand position at p100. Obviously, these actions can be sufficiently distinguished by considering the entire trajectory, but discerning them by analyzing only key frames is a challenge. In particular, the similarity in poses at p100, which is the most critical key frame, had a large influence on the reduced recognition accuracy.

In contrast, high recognition accuracy was achieved for the rest of the action classes, which had little visual similarity. Abchagi (front kick, A8) is the only kick motion, and it achieved the highest and second-highest recognition accuracies for the side and the front views, respectively. This is because it involves unique lower-body movements that can be clearly distinguished from the other actions. A1, A2, and A3 also have distinct visual characteristics. Arae makki (low block, A1) and sonnal makki (knife hand block, A3) have huge differences in the movement of the arm and the final pose at p100. Eolgul makki (high block, A2) is a unique motion in which the arms are placed above the subject’s head horizontal to the ground, as shown in Figure 8. Due to these characteristics, A1, A2, A3, and A8 all had high recognition accuracies above 90% with the side view, and they had higher recognition accuracies than other actions with the front view. Eolgul makki (high block, A2) was unique because it achieved a high recognition accuracy with the front view, while most of the other action classes obtained a high recognition accuracy with the side view. This may be because the arms blocked the face when they were above the head or horizontal, which made the features of this action class more apparent from the front view.

Several studies have used multi-view images that were captured simultaneously using multiple cameras for improving the recognition accuracy [32,38]. Although TUHAD provides images captured in two camera viewpoints, the recognition accuracy of the multi-view images cannot be evaluated because the action samples of the front and side views were captured separately. This is one limitation of TUHAD.

### 4.4. Backgrounds

The results of cross-background test show lower recognition accuracy compared to the test result of the model trained with both backgrounds as represented in Figure 8. This results clearly demonstrates that background feature was reflected in the training process and affected the resulting recognition accuracy. In the case of a model that trained with both backgrounds, there was no significant difference in recognition accuracy between two background samples, even though the number of samples with that background A is more than twice of the background B. This implies that features other than actions can be intervened in the training process even when a high-performance feature extractor such as CNN is used in the action recognition process. However, at the same time, it is possible to prevent the model becoming overfitted to secondary features like background by adding a few different types of samples to the dataset.

### 4.5. Comparison with Previous Recognition Algorithms

Table 10 shows the comparison result between recognition algorithms. The proposed method trained with TUHAD achieved the higher accuracy and less computation time compared to the previous taekwondo action recognition algorithms. However, as mentioned above, the previous recognition algorithm is specialized in their own dataset, some degree of accuracy decline was inevitable. The recognition algorithm proposed by Seo at al. [39] has a simple structure that calculates the image histogram and classifies it. However, due to the limitations of the method using simple histogram, it seems that the accuracy decreases in the dataset with diversity of brightness and subjects. Kong et al. [40] recognized the actions of taekwondo in the broadcast image with high accuracy. However, it also showed a limitation that it cannot be used in an action class that is not defined preliminarily. For this reason, only one input image was used in the performance evaluation without motion sequence information. For this reason, the accuracy of this method was evaluated relatively low compared to the value presented in their paper. However, the recognition accuracy improvement through the de-noising technique with modified SPOT was remarkable. Despite the additional computational time, it seems that it can be adopted to the proposed algorithm to improve recognition accuracy.

## 5. Conclusions

In this study, TUHAD was established to provide data for accurate vision-based action recognition of taekwondo. TUHAD contains 1936 samples representing eight fundamental unit techniques performed by 10 taekwondo experts who were recorded from two camera views. A key frame-based CNN classifier was developed, and the recognition accuracy was analyzed according to the image modality, key frame, and camera view. The results demonstrated that the CNN classifier achieved a recognition accuracy of up to 95.833%. TUHAD and the proposed classifier can contribute to advancing research regarding taekwondo action recognition, and the results on the optimal image capture setup can have broader applicability. For example, this information may also be applicable to other situations entailing the recognition of rapid actions outside the range of normal human movements. However, a critical prerequisite for applying the proposed method to action recognition is that the beginning and ending frames of the action (i.e., action detection) should be given prior to action classification. This will be explored in following research papers. TUHAD will be expanded to include more unit techniques as well as combinations of various unit techniques to recognize more complex and diverse taekwondo actions. In addition, more advanced action recognition and detection algorithms, such as trajectory-based and multi-view conditions, will be investigated.

## Figures and Tables

**Table 1:** The number of acquired action samples by subject and camera view.

View	T1	T2	T3	T4	T5	T6	T7	T8	T9	T10	Total
Front	96	100	96	100	88	100	96	104	100	96	976
Side	96	96	96	104	84	96	96	96	96	100	960

**Table 2:** Details of the structure of the key frame-based CNN.

Layer Index	Conv1	Conv2	Conv3	Conv4	FC1	FC2	Total
Output shape	58 × 58 × 16	28 × 28 × 32	13 × 13 × 64	5 × 5 × 128	3200 × 1	128 × 1	-
# of parameters ^1^	3216	4640	18,496	73,856	409,728	1032	514,168

^1^ Number of parameters from the training model using the RGB+D modality and p50 + p100 key frames.

**Table 3:** Types and number of training and test samples.

Camera View	Action Samples	Flipped Samples	Validation Samples	Training Samples	Test Samples
Front	976	1952	352	1404	196
Side	960	1920	343	1382	192

**Table 4:** Recognition accuracy with different image modality combinations.

Modality	Accuracy (%)	
	Front View	Side View
RGB	85.7	89.5
Infrared	77.0	80.2
Depth	83.1	93.7
Infrared + Depth	77.0	84.3
RGB + IR	82.1	88.5
RGB + D	87.7	94.2
RGB + IR + D	77.5	83.3

**Table 5:** Recognition accuracy according to key frames.

Number of Key Frames	Key Frames Used				Accuracy	
					Front View	Side View
2	p25	p50			81.1	90.6
	p25		p75		74.4	81.2
	p25			p100	85.2	91.1
		p50	p75		78.5	83.8
		p50		p100	88.2	95.8
			p75	p100	80.6	89.2
3	p25	p50	p75		77.5	86.9
	p25	p50		p100	89.7	95.3
	p25		p75	p100	80.1	90.6
		p50	p75	p100	88.2	92.7
4	p25	p50	p75	p100	87.7	94.2

**Table 6:** Average recognition accuracy by key frames.

Key Frame	Accuracy				Total Average Accuracy
	Two Key Frames		Three Key Frames		
	Front View	Side View	Front View	Side View	
p25	80.2	87.6	82.4	83.4	85.2
p50	82.6	90.1	85.2	85.9	87.2
p75	77.8	84.7	81.9	81.5	83.5
p100	84.6	92.0	86.0	87.5	88.9

**Table 7:** Recognition accuracy according to the camera view and action class.

Action Class	Accuracy (# of Test Samples)	
	Front View	Side View
A1	83.9 (398)	94.6 (408)
A2	91.1 (414)	90.2 (396)
A3	86.9 (374)	92.7 (356)
A4	74.0 (396)	87.1 (426)
A5	74.3 (447)	87.8 (395)
A6	80.6 (443)	83.9 (436)
A7	74.1 (432)	81.9 (437)
A8	91.1 (428)	93.4 (410)
Average	81.9 (3332)	88.8 (3264)

**Table 8:** Recognition accuracy according to the backgrounds.

Training Samples	Test Samples	Accuracy (%)	
		Front View	Side View
Background A	Background B	44.2	47.4
Background B	Background A	30.0	30.7
Background A + B	Background A	85.3	93.7
Background A + B	Background B	88.3	94.1
Background A + B	Random	88.3	95.8

**Table 9:** The comparison between datasets containing sports and martial arts.

Datasets	Field	Recording Environment	Modality	# of Classes (Martial Arts)	# of Subjects	# of Samples
MAHD [27]	Routine human actions, including some sports	Studio captured	RGB-D, Skeleton, IMU	27 (0)	8	861
UCF Sports Action Dataset	Overall sports	Broadcasting video	RGB	10 (0)	150	150
TTStroke-21 [11]	Sports (table tennis)	Video with similar camera position	RGB	20 (0)	129	129
MADS [32]	Martial arts, dancing, sports	Studio captured	RGB-D, Skeleton	36 (12)	5	216
TUHAD	Martial arts (taekwondo)	Studio captured	RGB-D, IR	8 (8)	10	1936

**Table 10:** The comparison with taekwondo action recognition algorithm.

Method	Accuracy (%)		Computation Time (ms)
	Front View	Side View	
Seo at al. [39] + Yolo V3/SVM	50.0	51.1	42.8
Kong et al. without SPOT [40]	52.1	56.8	16.4
Kong et al. with SPOT [40]	60.7	81.3	48.4
Proposed method	85.4	89.8	7.57

## Supplementary Materials

The following is available online at http://gofile.me/6AOtN/UePI6h55H, Data S1: Taekwondo Unit technique Human Action Dataset (TUHAD).
